# Semi-Annual Reunion of the Seventh and Eight District Dental Societies of the State of New York

**Published:** 1873-02

**Authors:** 


### PROCEEDINGS OF SOCIETIES.

### Semi-Annual Reunion of the Seventh and Eight District Dental Societies of the State of New York.

The Seventh and Eight District Dental Societies cover nearly the ground which was occupied by the old Western New York Dental Association. Upon the formation of the new societies this territory was, in accordance with the law, divided; and at the last meeting of the old association bitter regret was expressed by many of its members, at the necessity which arose of sundering the strong ties, which so closely bound heart to heart. New professional families were to be formed, and the old familiar faces were to be seen no more, only as occasional visitors at meetings when formerly they had been loved and honored members. So earnest seemed the desire to meet occasionally as brethren that provisions were made in the by-laws of each of the new societies, for the holding a semi-annual meeting together, alternately in the seventh and eight districts, and providing that in whichever district it should be held, the officers of that society should preside at the union meeting, and its members pay all the necessary expenses.

The Fourth Annual Reunion was held in the city of Rochester, commencing on Teusday, October 29, and was called for three days. The union of two district societies, the members of which are bound together by such strong fraternal ties, makes a large meeting; and as there is very little business to be transacted, the time is always wholly devoted to the legitimate objects of the gathering. Great credit is due to the officers and members of the Seventh District Society, more especially to its Secretary, J. Edward Sine, D. D. S., for the untiring labors which made of this one of the most pleasant and profitable sessions ever held in western New York.

The meeting was held in the Common Council Chamber, at the Court House, and was called to order by the President of the Seventh District Society, Dr. A. G. Coleman, of Ca[illegible] nandaigua.

Dr. Callender, of Toronto, President of the Ontario Dental Society, with Dr. Chittenden, of Hamilton; Dr. Relyea, of Belleville, and Dr. Wolverton, were presented as delegates from the Canadian Provinces, and were duly voted all the privileges of the floor.

The first paper read was entitled "Dental Progress," by Dr. E. S. Holmes, of Grand Rapids, Michigan. It was an able article, but the discussion brought out little that was new.

Leon F. Harvey, M. D., of Buffalo, then read a paper upon Facial Neuralgia, which called out an animated discussion.

Dr. Bristol—Said that he had been in the habit of making some simple local application, and then discharging the patient. But lately, he had himself been suffering from that distressing malady, and now he had a more positive knowledge of its horrors, and felt called upon to give his patient the most careful attention.

Dr. Chittenden—Thought its treatment was almost entirely experimental.

Dr. Barret—In answer to a question, said that of all the diseases to which we as dentists were called upon to minister, facial neuralgia was one of the most difficult to diagnose. It might arise from local irritation, or from constitutional or functional disturbances. It was an affection of the sensor filaments of the nerve, and should the cause be back of the Casserian ganglion, it might be difficult or impossible to cure. He should first search carefully for any local disturbance, and if there be such, remove the irritating cause. Sometimes periosteal inflammation is the cause of the pain, and in such cases would prescribe murcurius. At other times the source of the trouble is from exostosis. If constitutional treatment be required, he commonly referred the patient to his medical adviser. He then introduced J. N. Anderson, M. D., of Rochester, and moved that he be accorded the privileges of the floor.

Dr. Anderson returned thanks for the honor, and in the course of his remarks upon facial neuralgia, said he thought it most commonly referable to constitutional causes, but from his never having paid particular attention to local irritations in the oral cavity, he presumed he might sometimes have been mistaken in his opinion, and that the advice of an intelligent dentist might frequently have assisted him in making out a difficult case. In females he had found uterine affections a prolific source of this disease.

Dr. Gifford—Said that he lately had a case, where one tooth after another seemed the seat of intense and continuous pain, and each in turn was extracted, only giving temporary relief. He finally scarrified the gum, and treated with aconite and iodine, but without a permanent cure. Each of the teeth as extracted showed a marked case of exostosis.

Dr. Barrett—Said that here, it seemed to him, the trouble was periosteal, and indicated mercurius. Exostosis was hypertrophy of the cementum, caused by mal-activity of the perio-dental membrane. From some cause it was overstimulated. We know that *mercurius vivus* has a specific action upon all periosteum. He should therefore in such cases prescribe it with entire confidence, his experience justifying him in doing so.

Dr. S. B. Palmer, of Syracuse, was introduced to the meeting, and voted all the privileges of the floor. He said that in exostosis he had generally found the alveolar process partially absorbed.

Dr. French—Had been accustomed in violent cases of neuralgia to use hypodermic injections of morphia.

Dr. Wilson—Said that in one case of neurlagia, which seemed at times to radiate from one locality, upon extracting the tooth, he found instead of exostosis an absorption of the root. Perhaps Dr. Barrett was right, and this was mal-activity in the other direction.

Dr. Callender—Said that after trying various remedies with only partial success, he had applied to practicing physicians, and found them as ignorant as were the dentists.

Dr. Sine—Said that such difficulties might sometimes be traced to cutting the third molars.

After considerable desultory discussion, the subject was declared closed.

Dr. S. B. Palmer, of Syracuse, then read a paper, entitled

"Dental Probabilities," and for which he received a unanimous vote of thanks.

The second day's session opened with miscellaneous business.

Dr. Southworth—Presented a sample of gold filling by means of Dr. Macks gold screws.

Dr. Coleman—Claimed the use of gold screws in filling teeth long before they were "invented" by Dr. Mack.

Dr. French—Had also used them some years ago, and showed that their application was not as new as their patentee claims. Some rather severe remarks were made concerning the patenting of principles and appliances, which are in some cases nearly as old as the profession, and the attempt to force those who have been using them for years to pay a royalty to some consciencceless individual, who obtained his "invention" at second hand.

A valuable paper upon "Dental Education," by Prof. Barker, of Philadelphia, was read, and drew out considerable discussion.

An essay upon "Abnormal Dentition," by Prof. Truman, of Philadelphia, was read, and elicited much applause.

On motion the discussion of this paper was postponed, to listen to Dr. Barrett's paper.

Dr. W. C. Barrett, of Warsaw, then read an essay upon "The Anatomy and Histology of the Deciduous Teeth," illustrated by diagrams upon the blackboard, and by infant and fœtal skulls and bones.

Dr. Wilson—Thought this subject should receive our attention, not only from the value of the paper but from the importance of the subject. To thoroughly understand how to treat diseases of the teeth, it is necessary for us to know their structure.

Dr. Bristol—Asserted that the dental practitioner will not meet with success in filling "chalky" teeth, without so changing the diet of the patient, as that the deficiency of the proper proximates principles shall be supplied by nutrition, Instanced the case of a young man formerly a patient of his, and whose teeth he found it impossible properly to fill, but upon whom a change of residence, habits, and diet, wrought such a difference, that subsequently when he fell into the hands of the author of the paper under discussion, the nature of his teeth had been so altered that they were successfully filled, and are to-day not only beautiful in appearance but sound and likely to remain so.

Dr. Coleman—Cited the case of one of his own children, and asked the essayist what he would do when, as in this instance, the cuspids were erupted entirely outside the arch.

Dr. Barrett—Said the discussion was getting outside the limits of his paper, but perhaps it was as well to give it this latitude, and replied, that from what he knew of the case, he presumed the child had inherited from one parent large teeth, and from the other a small though perhaps well-shaped jaw.

Dr. Coleman—Remarked that this was the case.

Dr. Barrett—Under such circumstances would extract some teeth; either a bicuspid or the sixth year molar.

Dr. Southworth—Advocates the spreading of the arch, and objected to the extracting of any teeth.

Dr. Wilson—Acknowledged the soundness of Dr. Barrett's theories generally, but objected to extracting the six year molar. He believed the majority of cases of irregularity was caused by premature extraction of the deciduous teeth.

Dr. Barrett—Said that this was a case where there manifestly was not room for all the teeth. The arch did not need spreading as it was now perfectly symmetrical, nor could its form be altered without detracting from, instead of adding to the proper regularity of the features. It then only remained to question which tooth to remove. He thought if the first molar be extracted early enough, the requisite space might be gained, and the wisdom tooth subsequently become a serviceable organ, which in such cases is so apt to be the source of trouble. He did not by any means recommend the promiscuous extraction of six year molars. We would however sacrifice them when the salvation of more important teeth depended upon it.

Dr. Relyea agreed with Dr. Barrett in extracting six year molars when they were manifestly imperfect, and stood in the way of the development of better teeth.

Dr. Daboll—Said that to be successful in the treatment of teeth it is necessary for the dentist to have charge of them, not only from earliest childhood, but his opinion and advice should be sought by the mother during gestation. He prescribed the lacto phosphate of lime when rapid disintegration of the teeth is going on.

Dr. French—Said that during gestation the morbid craving of the appetite was sometimes uncontrollable, and required very judicious advice, not only from the dentist but from the physician.

Dr. Barrett—Remarked that chemical doctors were a failure the world over. It required something more than bricks and mortar to build a house. Vitality, the great architect which dominates the whole process, will not take the materials ready made, but must prepare them in her laboratory—the human system—specially for each case. All we have to do is to furnish the material in the food. In anything like a normal condition the appetite craves just what is needed to build up tissue. Pregnancy is in one sense an abnormal condition; yet he would allow the utmost possible limit to the appetite of the expectant mother. The diet must not consist of wholly farinacious food. Those articles must be taken, from which can be assimilated such principles as are needed to supply the imperative wants of the fœtus, that in its famishing condition it may not prey upon and rob the system of the mother.

Dr. Chittenden—Agreed with the previous speaker, that the phosphates were of but little service when administered directly, as they are not properly assimulated. He recommended plain diet, coarse food, milk, and that best of all foods, oatmeal.

The subject was energetically discussed at length by Drs. Callender, Edington, Wolverton, and others.

It was voted that immediately after the adjournment of the session the society accept the invitation of Mr. Powers, to visit the observatory connected with his block, which was done to the great pleasure of the members.

The afternoon session opened with a paper upon "Diseases of the Gums," by H. S. Miller, of Rochester.

Dr. Walter—Thought that tobacco had a malign influence upon the gums, and was frequently the cause of their puffy, turgid, inflamed condition. The necotine of the tobacco was a virulent poison, and we saw its effects plainly in the universally unhealthy condition of the tobacco-chewer's mouth.

Dr. Barrett—Said in answer to a question, that he apprehended that the main cause of the difficulty in such cases was the necessarily filthy condition of the mouth of the tobacco user. Especially might such appearances be seen about the place where he usually carried his "quid." The tissues of the mouth became so habituated to the action of the nicotine of the tobacco, that like the stomach of the confirmed arsenic eater, poisonous symptoms were only developed upon leaving off the habit.

Dr. Woodward—Said that although now a man of middle life, his wisdom teeth had never been erupted, and although not relative to the subject under discussion, he would like to inquire what was the probability of their yet coming.

Dr. Barrett—Said that perhaps when he got to be an old man, and all his other teeth were gone, the missing wisdom teeth might make their appearance, and then every one would wish to be gaping into his mouth, to see a remarkable case of third dentition. All the newspapers would herald it, and he would become famous.

The discussion was continued by Drs. Bristol, Chittenden, Edington, Coleman, Requa, and others.

An essay upon "Sections and Sectional Fillings," was read by Dr. J. Edward Sine, of Rochester. It was a demonstration of the advantages of what is commonly denominated cylinder filling; and elicited considerable discussion of the advantages and disadvantages of this system of performing operation, but nothing especially new was brought out.

An opportunity was given for the exhibition of appliances.

Dr. Southworth—Exhibited a new operating stool, with movable foot rest, intended to supply a want long felt, of a convenient stool upon which the operator may sit while at work over the chair.

Dr. Requa exhibited the Coraline base for artificial teeth.

Dr. Lewis exhibited the Elliott suspension burring engine.

Dr. Woodward exhibited a new automatic plugger, to be attached to any of the burring engines in use.

Some other appliances were exhibited, but nothing which was new.

Drs. Barrett, Daboll, and Southworth, of the Eight District, and Drs. Walter, Coleman, and Wilson, of the Seventh, were appointed visiting delegates to the Canada Dental Society.

A hearty vote of thanks was tendered to Profs. Barker and Truman, of Philadelphia, and to Dr. E. S. Holmes, of Michigan, for the valuable papers which they respectively had contributed to instruct and interest the dentists present, and the Secretary was instructed to convey the same to the gentlemen named.

The evening was devoted to the use and study of the microscope, and a lecture upon the subject was delivered by Charles Forbes, of the Rochester Free Academy. At its close some time was devoted to the examination of sections of bone, enamel, dentine, cementum, and of abnormal growths of the same, some prepared and mounted by Dı. Forbes, and some by Dr. Barrett.

At the close of the session the meeting adjourned, to meet in Buffalo, the last Teusday in October, 1873.

